# Perforated Duodenal Ulcer Post-Roux-en-Y Gastric Bypass: A Case Report

**DOI:** 10.7759/cureus.35112

**Published:** 2023-02-17

**Authors:** Lauren Hughes, Maryam Morris, Bryton Que, Geetan Rai, Juaquito Jorge, Frederick Tiesenga

**Affiliations:** 1 Medical School, Saint James School of Medicine - Anguilla Campus, The Quarter, AIA; 2 General Surgery, West Suburban Hospital Center, Oak Park, USA; 3 Surgery, Community Medical Center, Chicago, USA; 4 Surgery, Avalon University School of Medicine, Chicago, USA; 5 General and Bariatric Surgery, West Suburban Hospital, Oak Park, USA; 6 General Surgery, West Suburban Medical Center, Oak Park, USA

**Keywords:** duodenal ulcer, roux-en-y, bariatric surgery, bariatric case report, bariatric surgery complications, gastric bypass surgery, roux-en-y gastric bypass (rygb), perforated duodenal ulcer

## Abstract

Obesity is a growing health concern worldwide, with bariatric surgeries such as gastric bypass providing an effective treatment choice. However, a rare complication of gastric bypass is a duodenal ulcer. Currently, there is no exact incidence of this complication, and only a few case reports have been published in the literature. Presented is a case of a 32-year-old patient, eight years status post gastric bypass, who was evaluated for surgical repair of a large anterior perforated duodenal ulcer. This case report explores the relationship between patient history and gastric bypass surgery in the case of duodenal ulcer formation and perforation, as well as the diagnostic difficulty and modalities for duodenal ulcers in post-gastric bypass patients.

## Introduction

According to the 2022 World Obesity Atlas, there may be a billion individuals worldwide living with obesity by the year 2030 [1]. Among the available treatment options, bariatric surgery successfully reduces both premature mortality and comorbidities at a population level [2]. Research has continued to demonstrate that bariatric surgery dominates in effectiveness as a top treatment for obesity [3]. Bariatric surgery typically encompasses a few main procedures, including laparoscopic sleeve gastrectomy, laparoscopic gastric bypass, and laparoscopic adjustable banding. The most recently available data show that laparoscopic gastric bypass is associated with a higher risk of postoperative complications than laparoscopic sleeve gastrectomy and adjustable banding [4].

Regarding postoperative complications after gastric bypass, some, such as an anastomotic leak, are more common than others, such as peptic ulcer disease. Specifically, a perforated duodenal ulcer is a rare but reported event that may occur in patients who have undergone gastric bypass surgery [5]. This complication can occur as an acute emergency or even develop many years after the initial surgery [6]. It is challenging to pinpoint the exact causal factor of duodenal ulcers, as patient history may also play a significant role. Current data demonstrate a clinically relevant risk of ulcers in patients with a history of *Helicobacter pylori* infection, smoking, and non-steroidal anti-inflammatory drug (NSAID) use [7]. Additionally, not only are perforated duodenal ulcers rare events, but they also come with a substantial degree of diagnostic difficulty. For instance, these ulcers do not ordinarily present with free air on diagnostic radiology, limiting the number of methods used for diagnostic imaging [5].

## Case presentation

A 32-year-old female with a past medical history significant for morbid obesity status post-Roux-en-Y gastric bypass (RYGB), fibromyalgia, asthma, and chronic abdominal pain presented to the emergency department with complaints of worsening acute severe abdominal pain following consumption of takeout food. The patient endorsed feeling feverish, chills, nausea, and sharp, throbbing abdominal pain localizing to the right lower quadrant (RLQ) since the consumption of takeout food from a Puerto Rican restaurant earlier in the day. The patient’s surgical history included RYGB eight years prior and one cesarean section. Since completing the bypass procedure, the patient noted always having underlying chronic abdominal pain, intermittent diarrhea, and constipation. The patient endorsed chronic nausea and vomiting and recently underwent esophagogastroduodenoscopy (EGD) with no significant findings. The patient denied any other gastrointestinal symptoms or taking any NSAIDs. The patient reported occasional alcohol consumption and a former smoking history of a pack per week for approximately 10 years prior to cessation since the birth of her daughter several years ago. The patient was afebrile, with all vitals within normal limits. On physical examination, the patient presented with a soft, non-distended abdomen with diffuse abdominal tenderness to palpation throughout the abdomen but most noticeable in the RLQ. No guarding or rebound tenderness was found on examination. The patient was writhing in bed and unable to sit still due to the severity of the pain. Labs on admission showed mild leukocytosis (white blood cell count: 12.9 K/mcL), slightly elevated lipase levels (205 U/L​​), mild anemia (hemoglobin: 10.5 g/dL), and liver function tests within normal limits.

**Figure 1:**
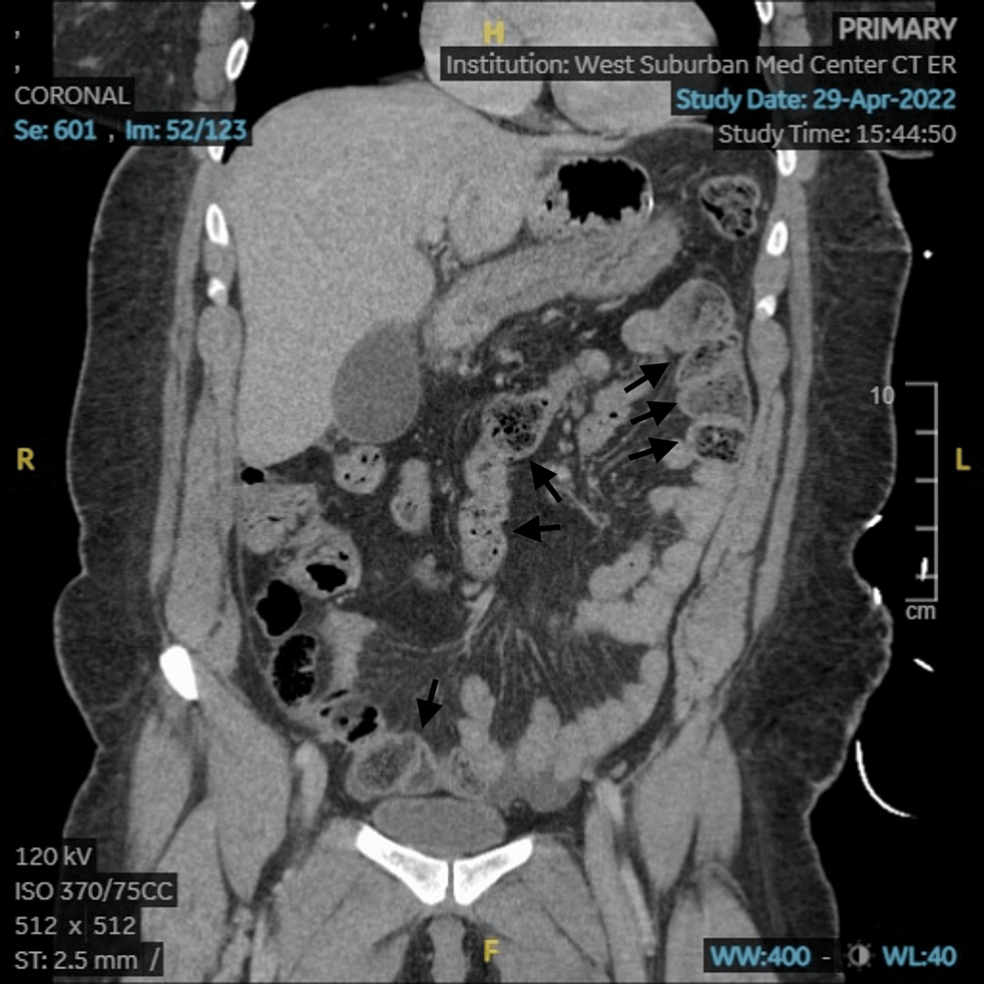
CT of the abdomen and pelvis with intravenous contrast coronal section showing mild circumferential distal small bowel wall thickening and mildly dilated loops of small bowel with feces

**Figure 2:**
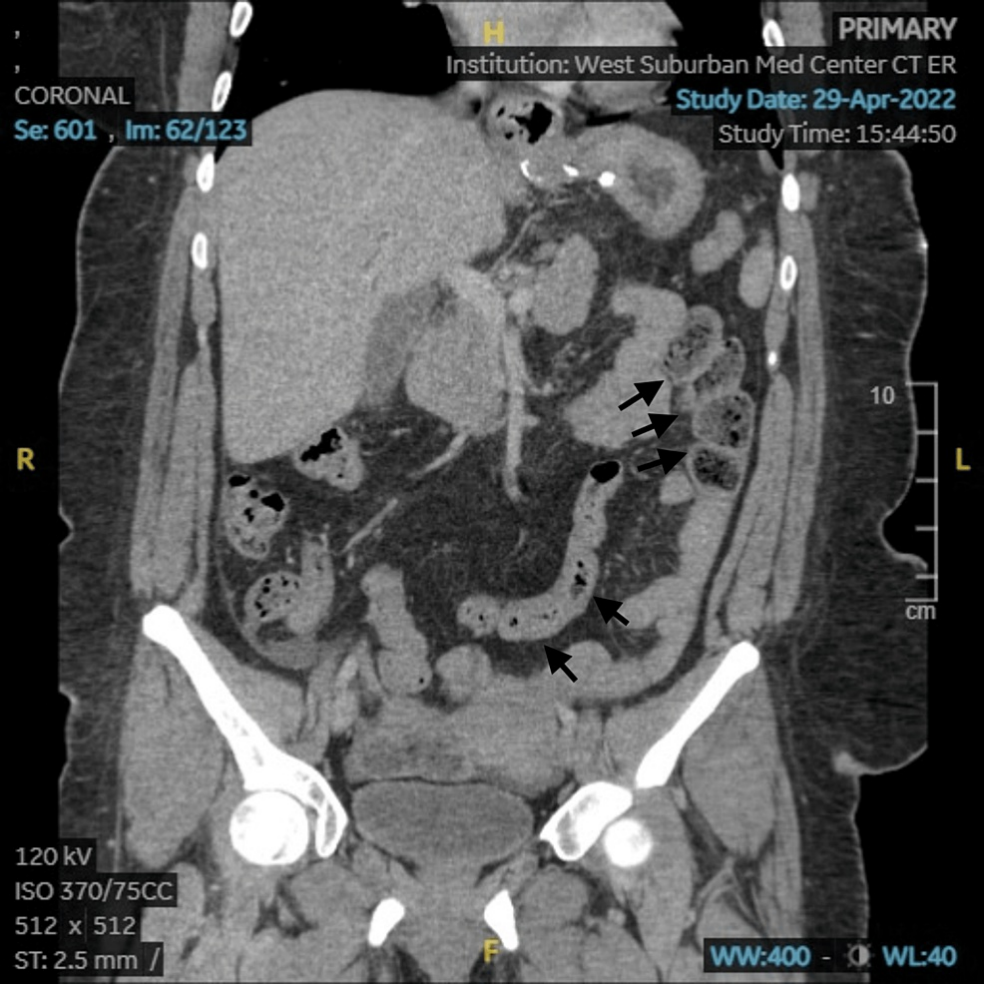
CT of the abdomen and pelvis with intravenous contrast coronal section showing mild circumferential distal small bowel wall thickening and mildly dilated loops of small bowel with feces

A computed tomography (CT) scan of the abdomen and pelvis with intravenous contrast was completed to rule out cholecystitis, appendicitis, and bowel obstruction. The scan revealed mild circumferential distal small bowel wall thickening compatible with mild enteritis, mildly dilated loops of small bowel with feces likely indicating stasis and likely a mild developing ileus, and mild gallbladder distention (Figures 1, 2). Due to the patient's right quadrant pain and gallbladder distention noted on her CT scan, further investigation with an ultrasound of the right upper quadrant was completed but revealed no evidence of acute cholecystitis. A transvaginal pelvis ultrasound was also completed to rule out a ruptured ovarian cyst. It revealed a small amount of pelvic free fluid but was otherwise normal and of no diagnostic significance.

**Figure 3:**
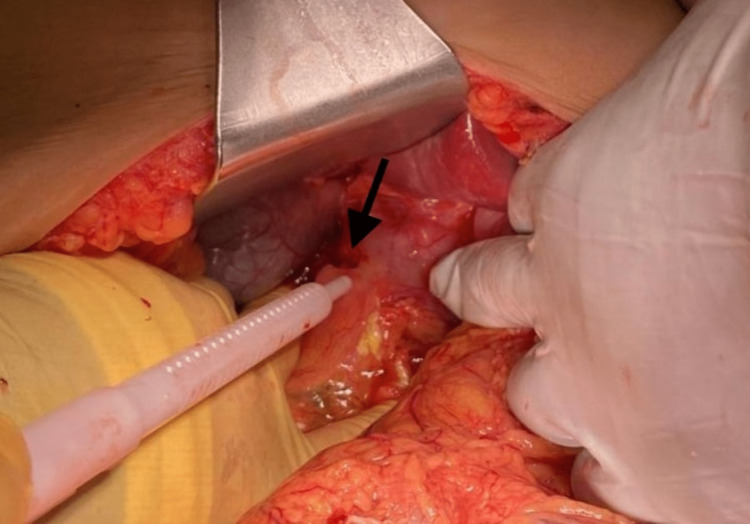
Large anterior hole in the duodenum with a perforated ulcer

The patient continued to endorse severe pain with little to no relief post intravenous morphine and ketorolac (Toradol). Henceforth, surgery was consulted, given the patient’s complicated history of gastric bypass and pain out of proportion to physical exam findings. Based on the patient’s history, physical exam findings, and imaging results, the patient underwent exploratory laparotomy for morbid obesity and acute abdomen, possible internal hernia. Upon entering the peritoneal cavity, a large amount of dark green bilious ascites was encountered during the procedure. There was no evidence of bowel obstruction. All limbs of the Roux-en-Y anastomosis were supple and very normal in appearance. During the exploration of the rest of the abdomen, at the level of the duodenum, there was a large anterior hole in the duodenum with a perforated ulcer (Figure 3). The ulcer was biopsied and sent to pathology, and the defect was repaired with a Graham patch without event. Meticulous hemostasis was assured, and antibiotic irrigation was utilized. A closed suction Jackson-Pratt (JP) drain was left in the area of the Graham patch, and the abdomen was closed anatomically.

Pathology findings of the biopsied duodenal ulcer consisted of a fragment of pink-tan soft tissue measuring 0.5 x 0.3 x 0.2 cm, verified as smooth muscle with full-thickness ulceration consistent with perforated gastric ulcer. Pathology also verified that the biopsy of the ulcer was negative for *Helicobacter pylori*. Blood culture and surgical culture showed no growth.

Postoperatively, the patient was transferred to the inpatient ward. The patient received and completed an intravenous piperacillin-tazobactam course for intra-abdominal prophylaxis, and a long-term proton pump inhibitor (PPI) regimen with pantoprazole was initiated. The patient was kept nil per mouth until started on a clear liquid diet on postoperative day four and then advanced to a low-fiber diet by postoperative day five, which was tolerated without difficulty. The patient's condition continued to improve, and a pain relief plan with hydromorphone and acetaminophen was adequate to control the patient's symptoms. On postoperative day six, the JP drain was removed, and the patient was discharged after an uncomplicated hospital stay. An outpatient follow-up visit one week later and a phone interview were conducted, during which the patient reported continued improvement in condition without significant complaints.

## Discussion

Duodenal ulcers are a rare, unexpected, and challenging-to-diagnose complication of RYGB surgery. Currently, there is no exact incidence of this complication, and there are only a few case reports published in the literature. Due to the anatomical change of the gastrointestinal tract in RYGB patients, there is more risk of marginal ulcers at the gastrojejunostomy than the duodenum [8]. In addition, patient history plays a significant role in diagnosing duodenal ulcers. For example, smoking, NSAID use, and *H. pylori* infection are known risk factors for duodenal ulcers [9]. However, the patient described only had a remote smoking history and did not have any of the other above risk factors. A possible cause of duodenal ulcers may be due to acid secretion from the remnant stomach due to hormonal and vagal stimulation [9]. Using long-term PPI in RYGB patients may be preventive for this complication. Although, the dosage of PPI may increase over time due to hypergastrinemia caused by a feedback mechanism [9]. Another possible cause may be bile reflux, which can damage the mucosa [5,9]. Other pathophysiology or risk factors may contribute to duodenal ulcers in RYGB patients. Therefore, physicians should consider duodenal ulcers in their differential diagnosis, irrespective of whether the patient meets the most known risk factors.

The diagnosis of duodenal ulcers is challenging due to the lack of signs on typical imaging modalities, which may rule out other causes. The patient described had no free air under the diaphragm and no sign of bleeding on the CT scan. Reviewing the literature, the lack of pneumoperitoneum was evident in other cases. This lack of pneumoperitoneum may be due to the preferential movement of air from the mouth to the stomach pouch through the jejunostomy rather than the biliopancreatic limb after RYBG [5]. The use of endoscopy to screen the duodenum for perforation is problematic due to the anatomical changes and is sometimes not possible [6]. Different types of imagining have been suggested in the literature to visualize the duodenum more effectively in RYGB patients. However, these methods will only be helpful if the physician considers duodenal ulcers in their differential diagnosis. For example, the patient described had an RYGB eight years prior, with no history of NSAID use or *H. pylori*. On imaging, there were signs of enteritis with no signs of perforation. These findings made duodenal ulcer diagnosis very difficult. It is essential to be aware of this rare complication. If a physician suspects a duodenal ulcer in RYGB patients, percutaneous gastrostomy, retrograde endoscopy, and virtual gastroscopy are some modalities to evaluate the duodenum [6]. Additionally, physicians aware of this rare complication and diagnostic difficulty may lower the surgical exploration threshold in this population.

## Conclusions

In presenting this case, the aim is to raise awareness of this rare complication in RYGB patients and for physicians to consider duodenal ulcers in their differential diagnosis. As other pathophysiology or risk factors may contribute to duodenal ulcers in RYGB patients, physicians should be mindful not to rely too heavily on the presence of risk factors for duodenal ulcers in patients before including duodenal ulcers in the differential diagnosis for RYGB patients. Furthermore, with the awareness that diagnosis of duodenal ulcer in RYGB patients is difficult, specifically via typical imaging modalities, the recommendation is for physicians to use percutaneous gastrostomy, retrograde endoscopy, and virtual gastroscopy as modalities to evaluate the duodenum as well as to consider surgical exploration more readily.
